# *Trichosporon asahii* Urinary Tract Infection in a Patient with Severe COVID-19

**DOI:** 10.1155/2021/6841393

**Published:** 2021-12-17

**Authors:** Verschoyle Cronyn, John Howard, Leslie Chiang, Lisa Le, Zandraetta Tims-Cook, Alida M Gertz

**Affiliations:** ^1^Wellstar Atlanta Medical Center Family Med Residency Program, 1000 Corporate Center Drive, Suite 200, Morrow 30260, GA, USA; ^2^Medical College of Georgia, Augusta, GA, USA

## Abstract

*Trichosporon asahii* is a yeast-like basidiomycete that is an emerging opportunistic infection in immunocompromised patients. Urinary tract infections due to *T. asahii* are rarely reported in the literature and typically seen only in immunocompromised patients. In addition to being immunocompromised, critically ill COVID-19 patients often have prolonged exposure to antibiotics, corticosteroids, and Foley catheters, which further increases their susceptibility to infection with *T. asahii.* There are limited case reports documenting successful treatment of *T. asahii* among hospitalized patients, particularly among COVID-19 patients, in the literature. Therefore, it is important that successful treatment regimens be reported. Here, we report a case of *T. asahii* urinary tract infection successfully treated with fluconazole and voriconazole in a 73-year-old male recovering from COVID-19. Urinary tract infections with *T. asahii* should be considered in persistently febrile COVID-19 patients with fungal urinary tract infections since prompt recognition and treatment can reduce the risk of disseminated disease and early mortality.

## 1. Introduction and Background

A growing body of literature has documented an increased risk of opportunistic fungal infections in patients infected with SARS-CoV-2, the virus that causes COVID-19 [1–5]. While candida is the most common fungal opportunistic infection recognized in the hospital setting, other fungal pathogens, such as *Trichosporon* species, are increasingly becoming recognized as an important cause of morbidity and mortality in the hospital setting [6, 7]. With the increasing number of COVID-19 cases, it is suspected that fungal opportunistic infections, including *Trichosporon*, will be more commonly encountered in the hospital setting.

*Trichosporon asahii* has been associated with a significantly increased incidence of infections over the past two decades [8]. This yeast-like organism is typically found in soil, plants, birds, bats and other mammals, decaying wood, bodies of water, certain types of insects, and also, in the human body's natural flora [9]. Infections resulting from *T. asahii* include superficial skin and hair infections, summer-type hypersensitivity pneumonitis, chronic pneumonia, urinary tract infections, meningitis, endocarditis, disseminated infection, and fungemia [8]. Although several of these infections are benign, they increase the risk for disseminated infections, which have been associated with fatality rates greater than 70%, largely due to high resistance rates to antifungal agents [6, 8].

Prior to the COVID-19 pandemic, *T. asahii* infections had been found to disproportionately affect immunocompromised individuals, including those with hematological malignancies, extensive burns, solid tumors, and AIDS [6]. Intravenous drug users and those undergoing peritoneal dialysis were also documented to be at an increased risk for *T. asahii* infection [6]. Although immunocompromised individuals are at greatest risk, serious infection in immunocompetent populations has been documented to occur as well. Infection with *T. asahii* has been found in immunocompetent individuals who have undergone ophthalmologic surgery and in patients with prosthetic devices including urinary catheters, endoscopic forceps, and arteriovenous grafts [6]. More recently, it has been suggested that COVID-19 and the conditions surrounding COVID-19, such as long duration in the intensive care unit (ICU), prolonged exposure to antibiotics and corticosteroids, and indwelling urinary catheters, may also increase the risk for *T. asahii* infection [2, 10]. A recent case report documented a COVID-19 patient admitted to the ICU who developed a triple pulmonary coinfection with *Pseudomonas* sp., *Stenotrophomonas* sp., and *Trichosporon* sp. [11]. Another recent study documented five cases of *T. asahii* fungemia in COVID-19 patients who were overexposed to antimicrobials and corticosteroids [10].

Despite the increasing emergence of *T. asahii* infections in the hospital setting and in COVID-19 patients, *T. asahii* urinary infections have rarely been documented in the literature. In the few documented cases of *T. asahii* urinary infections, the authors have described concerning complications including renal damage, potential dissemination, and increased mortality [9, 12].

More literature regarding the pathogenesis and treatment of *T. asahii* is needed given the risks associated with infection. In addition, in vivo studies of antifungal efficacy are limited and clinical guidelines regarding its treatment are sparse, resulting in challenges for clinicians when treating these infections in hospitalized patients. We report here a case of *T. asahii* urinary infection in a patient hospitalized for COVID-19, with multiple risk factors for *T. asahii* infection including long ICU duration and prolonged antibiotic therapy, who received multiple invasive medical devices during his hospital course and was discovered to have a probable malignancy. Furthermore, we document successful treatment of this infection and demonstrate the prevention of this infection from progressing to disseminated disease.

## 2. Case Presentation

A 73-year-old male with a past medical history of chronic obstructive pulmonary disease, hypertension, and cerebrovascular accident with a right-sided deficit and speech deficit presented in a somnolent state to the emergency room. The patient reportedly had generalized body aches, dyspnea, and cough, which had been progressively worsening over the past two to three days. The patient tested positive for SARS-CoV-2.

The patient experienced a prolonged hospital course, remaining in the hospital for approximately three months. A brief overview of the first month of hospitalization is given as follows: the patient was started on dexamethasone, azithromycin, and ceftriaxone at admission. His condition was complicated by a gastrointestinal bleed requiring multiple blood transfusions and a pulmonary embolism. The pulmonary embolism could not be adequately treated with anticoagulation due to his gastrointestinal bleed. His respiratory status declined, due to COVID-19, necessitating intubation which the patient required for most of his hospital stay. Broad-spectrum antibiotics were continued for the majority of his hospital course, switching to vancomycin, piperacillin-tazobactam, and levofloxacin to cover for ventilator-associated pneumonia. The patient required two courses of triple antibiotic therapy to cover for ventilator-associated pneumonia due to persistent leukocytosis and a chest X-ray significant for signs of infiltrates which remained following his initial treatment course for ventilator-associated pneumonia.

**Figure 1:**
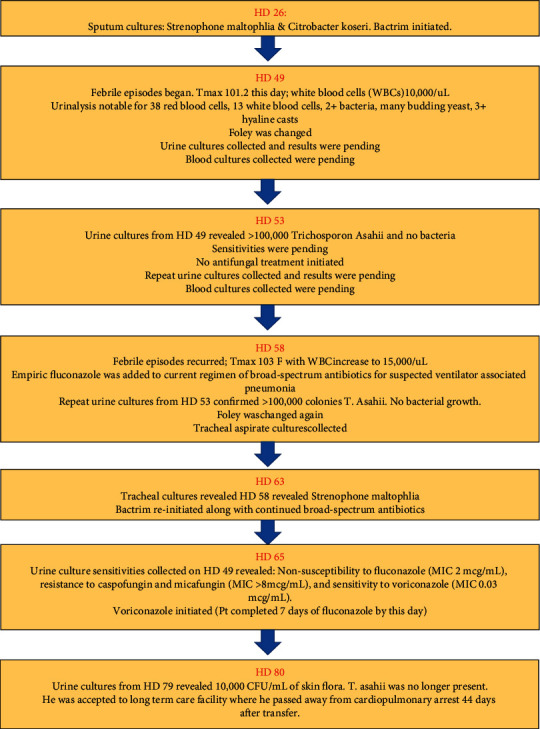
Flow chart outlining the timeline of events surrounding *T. asahii* infection, treatment, and resolution.

During the second month of his hospital stay, after his initial antibiotics courses had been completed, due to worsening respiratory status, sputum cultures were performed on hospital day (HD) # 26 which revealed *Stenotrophomonas maltophilia* and *Citrobacter koseri*, which were treated with trimethoprim-sulfamethoxazole (Figure 1). Due to his gastrointestinal bleed, the patient underwent an esophagoduodenoscopy and a colonoscopy. Esophagoduodenoscopy revealed mild antral gastritis with a deformed duodenal bulb as well as some mild diverticulosis. Colonoscopy revealed a large ascending-colon mass. Two biopsy samples were taken of this colon mass, which returned consistent with tubular adenoma without cytologic atypia. However, it was suggested by the pathologist that if this biopsy material was part of a larger lesion, a more advanced malignant lesion could not be excluded. The patient was too unstable during his hospitalization to undergo resection of the mass. On HD # 38, the patient received a tracheostomy which he tolerated well without complications. On HD # 49, the patient experienced a febrile episode (maximum temperature of 101.2 °F). At this time, the patient had both a Foley catheter and an internal jugular central line in place (he required both of these for the majority of his hospital course). Urinalysis, urine cultures, blood cultures, and a chest X-ray were ordered on the same day (Figure 1). Urinalysis revealed 38 red blood cells per High Powered Field (HPF), 13 white blood cells/HPF, 2+ bacteria, many budding yeast, and 3+ hyaline casts (Figure 1). Due to the bacteria and yeast in his urine, the Foley catheter was replaced the same day. Leukocytosis was only mild (10.6 cells per cubic millimeter of blood), but continued to increase to 15 cells per cubic millimeter of blood over the ensuing four days (Figure 1). The urine culture that resulted on HD # 53 revealed *T. asahii*. Isolates of *T. asahii* were obtained by performing a culture on a TSA blood agar plate with 5% sheep blood. The isolates were incubated at 35°C in an incubator at an ambient atmosphere for 24 hours. Isolates were identified using the VITEK 2 system (bioMérieux, Inc., Hazelwood, MO). Although the urinalysis had shown 2+ bacteria, no bacterial growth was seen in the urine culture. Blood culture revealed no growth after five days. Serum beta-D glucan was negative. The urine culture was sent to an outside laboratory (Quest Diagnostics Nichols Institute, Chantilly, VA) to determine sensitivities.

At Quest Diagnostics, the isolates were reincubated on Sabouraud Dextrose Agar (SDA) overnight. The inoculum was prepared in sterile demineralized water and standardized to a 0.5 McFarland. Twenty microliters of the suspension was added to a tube with 11 mL of Sensititre YeastOne Inoculum Broth and then prepared by inoculating 100 uL per well. In vitro advanced fungal susceptibility testing was performed using anidulafungin, micafungin, caspofungin, 5-flucytosine, posaconazole, voriconazole, itraconazole, fluconazole, and amphotericin B. *Trichosporon* organisms were tested with these agents using broth microdilution according to the Clinical and Laboratory Standards Institute (CLSI). The stock solutions purchased by Quest Diagnostics are prediluted (information regarding dilution preparation is not available). Isolates were identified using the Sensititre Trek YeastOne panel (Trek Diagnostic Systems, Cat. No. YO-9), which is a broth microdilution panel. The manufacturer's instructions were followed for setting up, reading, and analyzing the assay. Plates were incubated at 33–35°C for 24 hours minimum. The Sensititre system monitors for colorimetric change. MIC values were determined by demonstrating the lowest concentration of drug that does not demonstrate a color change. CLSI document M27 was used for the interpretation of results.

Pending sensitivity results, a repeat urine culture was ordered (HD # 53). On HD # 58, the patient again experienced a febrile episode, with a maximum temperature of 103.0°F accompanied by a leukocyte increase from 10 to 15 cells per cubic millimeter of blood (Figure 1). On HD # 58, the repeat urine culture results confirmed the presence of T. *asahii*. Again, no bacterial growth was seen in the urine culture. Antifungal medication had not been started initially as there was concern that the initial urine culture obtained on HD # 49 had revealed a contaminant or was reflective of asymptomatic colonization. However, with the repeat urine culture revealing T. *asahii*, the febrile episode, and leukocyte increase despite broad-spectrum antibiotics, antifungal coverage was deemed necessary. The patient was started on empiric fluconazole on HD # 58, and his Foley catheter was again replaced that same day (Figure 1). A tracheal aspirate culture was ordered which returned results on HD # 63 revealing *Stenotrophomonas maltophilia*. Treatment with trimethoprim-sulfamethoxazole was again started (Figure 1). On HD# 65, the sensitivities for the urine culture (sent to an outside laboratory on HD # 52) returned which revealed nonsusceptibility to fluconazole (MIC 2 mcg/mL), resistance to caspofungin and micafungin (MIC > 8 mcg/mL), and sensitivity to voriconazole (MIC 0.03 mcg/mL). Based on the advanced fungal testing, voriconazole was started on HD # 65 (following seven days of fluconazole) per infectious disease recommendations and continued for seven days (Figure 1). The patient had remained afebrile with improved leukocytosis since starting fluconazole (white blood cell count decreased from 15 cells per cubic millimeter of blood to 9 cells per cubic millimeter of blood). With the initiation of the voriconazole, the leukocyte count continued to drop to normal levels (4.40 cells per cubic millimeter of blood).

A percutaneous endoscopic gastrostomy tube was placed on HD # 78, and the patient tolerated the procedure well without complication. Urine cultures were obtained on HD # 78 and revealed 10,000 CFU/mL of usual skin flora. *T. asahii* was no longer present in urine (Figure 1). At this point, the patient was accepted and transferred to a long-term care facility on HD # 80. At this long-term care facility, multiple attempts failed to fit the patient with an aerosolized tracheostomy collar. A decision was made to initiate palliative care. On HD # 44 at the long-term care facility, he was found without spontaneous respirations or palpable pulse.

## 3. Discussion

This case adds to the growing literature documenting opportunistic infections in COVID-19 patients. Our case also contributes to the literature suggesting opportunistic fungal infections such as *Trichosporon* may be increasingly encountered in patients with severe COVID-19 [13, 14]. The occurrence of these coinfections is important to elucidate as it highlights another difficulty of managing patients with severe COVID-19. In addition to the complexity of treating this potentially fatal virus, clinicians must also be aware of bacterial and fungal coinfections that may further complicate illness.

Bardi et al. examined over 100 patients with COVID-19 in the ICU setting for the development of coinfection. This study found that nearly half of the patients developed a bacterial or fungal coinfection and that these infections were associated with a high risk of mortality [13]. Although the overall incidence of *T. asahii* causing UTIs is not well elucidated, a study by Li et al. that examined *T. asahii* infections in Asia, Europe, North and South America, and Africa found the four greatest risk factors for infection included antibiotic use, invasive medical devices, neutropenia, and ICU hospitalization [8]. In this study, it was observed that bloodstream infections were the most common, followed by urinary infections, respiratory infections, and infections of the integumentary system [8].

*Trichosporon* infections have been associated with mortality rates ranging from 15 to 70% [6, 8]. This is explained in part by high resistance rates to antifungal agents, which in turn are due, partially, to the poor ability of antifungal agents to penetrate biofilms produced by this organism [6]. Another significant factor that contributes to antifungal resistance is previous or prolonged exposure to azole agents [15]. Despite the decreased effectiveness of voriconazole in treating *T. asahii* infections, the European Society for Clinical Microbiology and Infectious Diseases and the European Confederation of Medical Mycology 2014 guidelines recommend voriconazole as the treatment of choice for trichosporosis over other antifungals such as fluconazole, amphotericin B, and caspofungin, in in vitro studies of antifungal resistance [6].

This report emphasizes the need for clinicians to minimize risk factors that predispose patients to develop these coinfections. These risk factors include prolonged exposure to antibiotics and corticosteroids and receiving invasive medical devices (such as Foley or central-line catheters). This case study documents a *T. asahii* urinary tract infection, along with the risk factors that may have contributed to this coinfection, and outlines the management approach used to treat this coinfection. A similar report by Segrelles-Calvo et al. (2021) documented a case of *T. asahii* pneumonia which occurred in a patient with COVID-19 [11]. The individual described in that report had many risk factors similar to those of the patient described here, including prolonged corticosteroid and antibiotic use and long ICU duration (28 days at diagnosis of *Trichosporon* infection) [11]. Segrelles-Calvo et al. also detail how the patient failed to respond to treatment with voriconazole and died on day 38 [11].

The authors in this report highlight our approach to isolating the *T. asahii* organism and how pathologic infection was differentiated from asymptomatic coinfection. This was achieved by replacing the Foley catheter and reculturing the urine which again revealed *T. asahii*. We also document the clinical course of the patient and the correlated improvement in symptoms and associated laboratory values once initiating antifungal therapy. We outline our treatment approach with empiric fluconazole, which, although advanced fungal sensitivity testing revealed nonsusceptibility to fluconazole (MIC 2 mcg/mL), we believe possessed a moderate degree of effectiveness against the *T. asahii* organism. Our rationale is supported by an article by Kahlmeter et al. who suggest that isolates that are found to be nonsusceptible do not necessarily possess a resistance mechanism [16]. Furthermore, improvement in the patient's clinical condition continued when an antifungal agent, to which *T. asahii* was susceptible, voriconazole (MIC 0.03 mcg/mL), was commenced. A recent study by Ahangarkani provides additional support for the high potency of voriconazole (GM MIC of 0.075 *μ*g/ml) against *T. asahii* [17].

## 4. Conclusions

This report serves to highlight a particularly severe type of coinfection involving a multidrug-resistant fungal pathogen linked to high mortality and with limited treatment options. It is proposed that early suspicion and detection of this organism may allow for more prompt initiation of antifungal medication (voriconazole), likely reducing the risk of disseminated disease and mortality. Physicians treating hospitalized patients with COVID-19 should be aware of the increased risk of fungal opportunistic infections, such as *T. asahii*. Early suspicion and detection of *T. asahii* may allow for earlier initiation of treatment, reducing the risk of progression to a more severe disseminated infection. As *T. asahii* is often resistant to a wide variety of antifungals, susceptibility testing may significantly impact patient outcomes by allowing more targeted treatment. Continued research investigating the diagnosis and treatment of *Trichosporon* infections is needed to provide clinicians with the knowledge of how to best manage these infections when encountered in the hospital setting.
